# Stomatopoda of Greece: an annotated checklist

**DOI:** 10.3897/BDJ.8.e47183

**Published:** 2020-03-26

**Authors:** Panayota Koulouri, Vasilis Gerovasileiou, Nicolas Bailly, Costas Dounas

**Affiliations:** 1 Hellenic Center for Marine Recearch (HCMR), Heraklion, Greece Hellenic Center for Marine Recearch (HCMR) Heraklion Greece; 2 WorldFish Center, Los Baños, Philippines WorldFish Center Los Baños Philippines

**Keywords:** Stomatopoda, Greece, Aegean Sea, Sea of Crete, Ionian Sea, Eastern Mediterranean, checklist

## Abstract

**Background:**

The checklist of Stomatopoda of Greece was developed in the framework of the LifeWatchGreece Research Infrastructure (ESFRI) project, coordinated by the Institute of Marine Biology, Biotechnology and Aquaculture (IMBBC) of the Hellenic Centre for Marine Research (HCMR). The application of the Greek Taxon Information System (GTIS) of this project has been used in order to develop a complete checklist of species recorded from the Greek Seas. The objectives of the present study were to update and cross-check all the stomatopod species that are known to occur in the Greek Seas. Inaccuracies and omissions were also investigated, according to literature and current taxonomic status.

**New information:**

The up-to-date checklist of Stomatopoda of Greece comprises nine species, classified to eight genera and three families.

## Introduction

Stomatopoda, also called "mantis shrimps or mantis prawns", is one of the most distinctive orders of Crustacea. They were well known from ancient times since Aristotle, the greatest of all naturalists, who described, for the first time in detail, the external morphology of the mantis shrimp *Squilla mantis* (Voultsiadou and Vafidis 2007, Voultsiadou et al. 2017). They are common members of benthic ecosystems in tropical and subtropical marine waters throughout the world (Müller 1994). However, only a small number of species are known from temperate seas (Ahyong 2012). Mantis shrimps construct burrows in soft bottoms or live in crevices and holes of hard substrates (Müller 1994). Their larval development occurs in the plankton (Özel 1985). They are amongst the most efficient crustacean raptorial predators, having unique adaptations for hunting their prey (Ahyong 2012). They are often caught by trawls in commercial shrimp operations. In some parts of the world, mantis shrimps are considered a delicacy and consequently are fished commercially and marketed (e.g. Japan, Vietnam, Philippines and China). In Mediterranean countries, only *Squilla mantis* and *Erugosquilla massavensis* reach marketable densities and constitute a target for local fisheries (Colmenero et al. 2009).

Taxonomy of stomatopods still causes several difficulties for non-taxonomic specialists who only want to determine their specimens within the scope of non-taxonomic studies. Fortunately, several revisions, reviews and re-descriptions are available for this group of animals, though numerous short publications are scattered over many journals (Müller 1994). Consequently, 450 species of stomatopods are known worldwide, arrayed in 7 superfamilies, 17 families and over 70 genera (Ahyong 2012). Amongst these, only 12 stomatopod species are known in the Mediterranean Sea after a few studies carried out in this region (Manning and Froglia 1979, Abelló et al. 1993, Dounas and Steudel 1994, Özcan et al. 2008, Colmenero et al. 2009, Bakır et al. 2014, Ounifi-Ben Amor et al. 2015). The only contribution to the Greek records of the taxonomic group of Stomatopoda has been made by Dounas and Steudel (1994). During several biological investigations along the continental shelf of Crete (1988-1991), an interesting collection of stomatopod crustaceans was obtained from soft substrates in depths ranging from 10 to 200 m. Since then, no new species records have been provided. Data on the occurrence and distribution of stomatopods in the Greek Seas remain scarce (Kevrekidis and Galil 2003, Dimitriadis et al. 2019).

The first attempt for developing a checklist of Stomatopoda was carried out within the framework of the "Greek Biodiversity Database" project, coordinated by the Aristotle University of Thessaloniki in Greece (2005-2008). In 2010, a database was set up online in order to record the occurrence of these marine species in the Greek Seas. The World Register of Marine Species (WoRMS Editorial Board 2020) and the European Register of Marine Species (ERMS, now part of WoRMS) created the reference of Koukouras (2010) in order to include the list of these marine species provided by the Greek Biodiversity Database during the European project PESI.

The aim of the present study was to provide an updated checklist of Stomatopoda of the Greek Seas. For this purpose, an older list of stomatopod species was updated and annotated according to recent literature and current taxonomic status of the species.

## Materials and methods

The annotated checklist of Stomatopoda of Greece was developed within the framework of the LifeWatch Greece Research Infrastructure (ESFRI) project and coordinated by the Hellenic Centre for Marine Research during the period 2013-2015 (Arvanitidis et al. 2016). Bailly et al. (2016) give the general principles used for elaborating the annotated checklist of Stomatopoda of Greece. The checklist of Stomatopoda was constructed, based on the classification and species records, listed as present in Greece and extracted from the dataset of WoRMS/ERMS for marine species (Koukouras 2010, Costello et al. 2020). Then, all relevant publications were reviewed and the species reported to date have been added to the list. All Stomatopoda records were cross-checked for their taxonomy in WoRMS (WoRMS Editorial Board 2020). The main synonyms are presented for each species under the "Nomenclature" field. Non-indigenous species are marked in the "Native status" field. Detailed information is also presented with regards to the distribution, bathymetric range and biotopes for each species in the Greek Seas and adjacent regions, along with key references, based on a thorough literature review.

## Checklists

### Checklist of Stomatopoda known to occur in Greek waters

#### Stomatopoda

F8A80A5A-D82D-5F30-B9E6-4C946A3F55CD

#### Nannosquillidae

CB1D81C6-0F7E-58EB-B770-9AC8FDE01BF4

#### Allosquilla africana

(Manning, 1970)

D8E7276A-77C6-505A-832F-4C6F1A6A4E53

Allosquilla adriatica Stevcic, 1979; *Allosquilla adriatica* Manning & Froglia, 1979; *Heterosquilla africana* Manning, 1970

##### Distribution

**E. Mediterranean**: Crete Island, Heraklion Bay, 160 m, clayey silt bottom (Dounas and Steudel 1994); Aegean coasts of Turkey, Sıgacık Bay, 200 m, muddy bottom (Bakır and Çevirgen 2012). **Adriatic Sea**: west of Pomo rocky islet, 130-150 m and west of Pomo Pit, 216-222 m, muddy bottoms (Manning and Froglia 1979, Froglia and Manning 1986). **W. Mediterranean**: Tyrrhenian Sea (Ranieri and Mori 1994, Colloca et al. 2004). **Atlantic Ocean**: off the Niger delta, Gulf of Guinea, 148-174 m (Manning 1977).

#### Nannosquilloides occultus

(Giesbrecht, 1910)

F805AA08-18A9-5889-A526-8D1D4CAE4BCC

Nannosquilla occulta (Giesbrecht, 1910); *Lysiosquilla occulta* Giesbrecht, 1910

##### Distribution

**E. Mediterranean**: Crete Island, Mirabello Bay, 20 m, silty sand bottom (Dounas and Steudel 1994); Aegean coasts of Turkey, Gulf of Edremit, 15-20 m, *Posidonia oceanica* meadow (Kocataş 1981); coasts of Israel, 40-50 m, muddy bottoms (Lewinsohn and Manning 1980). **Adriatic Sea**: 34 miles off Fano, 60 m (Manning and Froglia 1979). **W. Mediterranean**: Gulf of Naples, Mergellina, 30 m (Steuer 1933). **Atlantic Ocean**: west African waters off Senegal, Sierra Leone, Congo and Angola, 30-200 m (Manning 1977).

#### Platysquilla eusebia

(Risso, 1816)

F79EC3DF-B846-574F-8B41-77B404B85C9E

Squilla eusebia Risso, 1816

##### Distribution

**E. Mediterranean**: Aegean Sea, Lesvos Island, 2.5 m, very fine sandy bottom (Udekem d'Acoz 1995); larvae of this species have been found along the Turkish coasts of the Aegean (Özel 1985); coasts of Israel, off Palmahim, 8 m, *Cymodocea nodosa* meadow (Galil 2004). **Adriatic Sea**: east coast of Istria Peninsula and SW of Opatija, in the first bay SW of Rt Kolova, 3-10 m, well-sorted very fine sandy bottoms (Abed-Navandi and Dworschak 1997). **Atlantic Ocean**: coasts of Portugal, France, west coast of Ireland and North Sea (Manning 1977, Lewis and Gittenberger 2013 and references therein).

#### Parasquillidae

B453FA9F-BCF2-5ACC-B748-3F0F4AC2490E

#### Parasquilla ferussaci

(Roux, 1828)

249ACD2C-48FC-50EB-B28D-34D21263E529

Squilla bruno Prestandrea, 1833; *Squilla ferussaci* Roux, 1828

##### Distribution

**E. Mediterranean**: Crete Island, Rethymno Bay, 50 m (Dounas and Steudel 1994); SE coasts of Rhodes, Haraki, 150 m, biogenic detritus mixed with mud and rocks (Corsini-Foka and Pancucci-Papadopoulou 2012); Lesvos Island, 130 m, sandy and coralligenous bottom (ELNAIS 2020); north Aegean Turkish coasts, Babakale, 150-200 m, sandy-silt bottoms (Özcan et al. 2008). **W. Mediterranean**: from Nice to Sicily, including the Gulf of Naples and Porto Santo Stefano, off Fuengirola (North Alboran Sea, muddy bottom), off Gavà (Catalan Sea, 95 m), Menorca (Balearic Islands, 174 m, muddy sand bottom) (Colmenero et al. 2009 and references therein). **Atlantic Ocean**: Gulf of Cadiz, off Portugal (252-550 m) and Portuguese coasts, Madeira Island, west African waters, including off Liberia coasts (190-220 m), Bissagos sand bank (180 m, Guinea Bissau), off Vridi (100 m), Ivory Coast, off W. Morocco (Manning 1977, Abelló and Maynou 2018, Colmenero et al. 2009 and references therein).

#### Pseudosquillopsis cerisii

(Roux, 1828)

01A2E14F-1DA0-5FC3-A626-8E373B775A9E

Squilla broadbenti Cocco, 1833; *Squilla cerisii* Roux, 1828

##### Distribution

**E. Mediterranean**: Aegean Sea, SE coast of Peloponnese (Guérin 1832). **C. Mediterranean**: Sicily (Abelló and Maynou 2018 and references therein). **W. Mediterranean**: Gulf of Naples (Giesbrecht 1910); Corsica, Algeria, southern coasts of France (Toulon, Gulf of Lions), Balearic Islands, off Vilanova (fish stomach contents, Catalonia, 10-20 m, *Posidonia oceanica* meadows) (Abelló and Maynou 2018 and references therein). **Atlantic Ocean**: off west African waters of Gorée (Senegal), port of Banana (Democratic Republic of the Congo) (Manning 1977 and references therein).

#### Squillidae

BA88D537-C715-5B3B-ADC0-A85A28F11D29

#### Erugosquilla massavensis

(Kossmann, 1880)

282871BE-B84E-561E-8BB4-E22E0F8922DB

Squilla massavensis Kossmann, 1880

##### Ecological interactions

###### Native status

Non-indigenous species

##### Distribution

**E. Mediterranean**: Aegean Sea, Saronikos Gulf (ELNAIS 2020); Crete Island, Malia Bay, 60 m, sandy-silt (Dounas and Steudel 1994); Rhodes Island, 20-29 m (Kevrekidis and Galil 2003); Karpathos Island (Corsini and Kondilatos 2006); Aegean coasts of Turkey, Sigacik Bay, 150-200 m, sandy-silt bottom (Özcan et al. 2008); Cyprus, Famagusta Bay, 27 m, muddy sand bottom (Lewinsohn and Manning 1980, Özcan et al. 2014); southern coasts of Turkey, Antalya Bay, 15-20 m, fine sand bottoms (Lewinsohn and Manning 1980 and references therein); coasts of Syria (Ounifi-Ben Amor et al. 2015 and references therein); coasts of Lebanon, St. George’s Bay (Lewinsohn and Manning 1980); coasts of Israel, 5-183 m, from near-shore sandy bottoms to offshore muddy bottoms; coasts of Egypt, Suez Canal and off Alexandria, 27 m, fine sand with little mud at bottom, *Amphioxus* ground bottom, Port Said, Lake Timsah (Lewinsohn and Manning 1980 and references therein); coasts of Libya, Tubruk, 5.5 m, sandy bottom (Shakman and Kinzelbach 2007). **Sea of Marmara**: 30-35 m, muddy bottoms (Katağan et al. 2004). **C. Mediterranean**: southern Ionian Sea, Zakynthos Island, 10 m, muddy bottom with sandy patches (Dimitriadis et al. 2019); Sicily, SE coasts, sandy-sandy mud bottoms, >20 m, (Corsini-Foka et al. 2017, Gianguzza et al. 2019); Malta, Valetta Grand Harbour, 12 m, fine sands and muddy sediment (Stern et al. 2019); coasts of Tunisia, Gulf of Gabès, 20 m, sandy-muddy bottom and Southern Lagoon, 3 m, muddy bottom (Ounifi-Ben Amor et al. 2015).

#### Rissoides desmaresti

(Risso, 1816)

D170D05E-B690-5998-9E1C-1DE116FF30DA

Meiosquilla desmaresti (Risso, 1816); *Squilla desmaresti* Risso, 1816

##### Distribution

**E. Mediterranean**: Crete Island, Agia Pelagia 15-18 m, coarse sand with biogenic detritus and Chania Bay (Dounas and Steudel 1994); Messiniakos Gulf (Guérin 1832); coasts of Cyprus, Famagusta, 36 m and Akrotiri, 54 m (Lewinsohn and Manning 1980); Izmir Bay, Turkish Aegean Sea (Kocataş 1981); southern coasts of Turkey (Gökçe et al. 2016); larvae of this species have been found along the Turkish coasts of the Aegean (Özel 1985); coasts of Israel, 40-57 m, sandy and muddy bottoms (Lewinsohn and Manning 1980). **Adriatic Sea**: Gulf of Venice, 27 m, offshore sands covered by a layer of "red-mud" from Aluminium plant; Conero (70 and 75-80 m), NE of Ancona (54 m, 73 m of muddy sands with *Cellaria* sp.) and NE of Fano (52 m, sandy mud bottoms) (Manning and Froglia 1979). **W. Mediterranean**: off Elba island, north Tyrrhenian Sea, 8 m, sandy bottom covered by *Cymodocea nodosa* meadow (Ranieri and Mori 1991); off Nice and Gulf of Naples (Manning 1977); Mallorca, Balearic Islands, 20 m, muddy sand; Sant Carles de la Ràpita, Catalonia, 18 m, terrigenous mud (Abelló et al. 1993). **Atlantic Ocean**: along the European coast north to southern England and the southern North Sea to a depth of at least 75-80 m (Manning 1977, Lewinsohn and Manning 1980 and references therein).

#### Rissoides pallidus

(Giesbrecht, 1910)

9620204A-DC48-5FA4-8A29-E7EF5F453E54

Meiosquilla pallida (Giesbrecht, 1910); *Squilla pallida* Giesbrecht, 1910

##### Distribution

**E. Mediterranean**: Crete Island, Heraklion Bay, 190 m, silty clay and off Georgioupolis coast, 105 m (Dounas and Steudel 1994); Rhodes Island, 63-85 m (Kevrekidis and Galil 2003); Turkish Aegean coasts, Datca Peninsula, 280 m, mud; Kusadası Bay, 105 m, mud; Sıgacık Bay, 550 m (Kocataş and Katağan 1995, Kocak 2011); coasts of Cyprus (Demetropoulos and Neocleous 1969); Israel, off Bardawil lagoon, 91.5 m and off Palmahim, 80 m, mud (Lewinsohn and Manning 1980). **Adriatic Sea**: Pomo Island, 115-132 m, 200 m, 215 m; Incoronata Island, 125 m; SW of Ludetta, 133 m, coarse sands; western Pomo pit, 220 m, muds; NW of Isole Tremiti, 104 m, sandy muds with empty shells of the gastropod *Monodonta coclear*; SE of Lagosta, 285 m, muddy sands (Manning and Froglia 1979). **C. Mediterranean**: coasts of Tunisia, 170 m and 200 m (Forest and Guinot 1956); Gulf of Patti, Sicily, southern Tyrrhenian Sea, 500 m (Manning and Froglia 1979). **W. Mediterranean**: Gulf of Naples (Giesbrecht 1910); off Elba island, northern Tyrrhenian Sea, 200-400 m (Ranieri and Mori 1991); Spain: Catalan Sea, 290-300 m (Valladares 1987); Catalonia, Vilanova, 110-247 m, terrigenous mud, Blanes, 110-113 m, terrigenous mud (Abelló et al. 1993). **Atlantic Ocean**: West African waters of Morocco (155 m and 160 m), Senegal (200-400 m), Ivory Coast (100-109 m), Morocco to Senegal (80-500 m) (Manning 1977, Lewinsohn and Manning 1980 and references therein).

#### Squilla mantis

(Linnaeus, 1758)

40C813B7-8986-5DD4-A6F9-7021BD92C42F

mantis Linnaeus, 1758

##### Distribution

**E. Mediterranean**: Aegean Sea, Peloponnese, Nafplion (Guérin 1832); Saronikos Gulf (Panagiotopoulos 1916, Athanassapoulos 1917); Crete Island, Malia Bay, 30 m, silty sand with *Caulerpa prolifera*; Rethymno Bay; Chania Bay; off Georgioupolis coast, 105 m; Ierapetra coasts, 70 m (Dounas and Steudel 1994); Rhodes Island, 31-49 m (Kevrekidis and Galil 2003); in numerous localities of the coasts of Egypt, Israel, Syria, Turkey and Cyprus in depths from 2 up to 112 m and muddy substrates (Lewinsohn and Manning 1980 and references therein). **Adriatic Sea**: off Fano fishing grounds, 6-16 m (Piccinetti and Piccinetti Manfrin 1970a, Piccinetti and Piccinetti Manfrin 1970b, Piccinetti and Piccinetti Manfrin 1971). **C. Mediterranean**: coasts of Tunisia, 22-90 m (Forest and Guinot 1956, El Lakhrach et al. 2012). **W. Mediterranean**: Gulf of Naples; Gulf of Lions; off Algeria, 80-280 m (Manning 1977, Lewinsohn and Manning 1980 and references therein). **Atlantic Ocean**: coasts of southern Europe, Canary Islands and West Africa from Morocco to southern Angola; shore to a depth of more than 200 m (186-247 m), generally in 120 m or less (Manning 1977).

## Discussion

Only twelve stomatopod species are known in the Mediterranean Sea, including three aliens of Indo-Pacific origin: the mantis shrimps *Erugosquilla massavensis* and *Clorida albolitura*, as well as the erythrosquillid *Erythrosquilla* sp., yet unidentified, that has been reported, based on a post-larval specimen collected from plankton in the Ligurian Sea (Colmenero et al. 2009). With the exception of *Squilla mantis*, *Rissoides pallidus* and, to a lesser degree, *Rissoides desmarestii*, which are rather commonly captured by trawling (Colmenero et al. 2009), studies concerning data on the occurrence and distribution of other stomatopods in this region are much fewer (e.g. Lewinsohn and Manning 1980, Ranieri and Mori 1991, Ranieri and Mori 1994, Dounas and Steudel 1994, Abelló et al. 1993, El Lakhrach et al. 2012, Bakır and Çevirgen 2012, Özcan et al. 2014).

The updated checklist of Stomatopoda of Greece includes a total of nine species, classified into eight genera and three families. Recently, checklists for marine crustaceans such as Cumacea, Mysida and Lophogastrida have also been published from the Greek Seas (Koulouri et al. 2016a, Koulouri et al. 2016b). As mentioned above, after Dounas and Steudel (1994), who studied the stomatopods along the continental shelf of Crete and therefore largely contributed to the Greek records of this taxonomic group, no new species records have been provided. Until recently, only scattered distribution data of already recorded stomatopod taxa have been published (e.g. Kevrekidis and Galil 2003, Dimitriadis et al. 2019). Moreover, further research is needed for the verification of the presence of the stomatopod species *Pseudosquillopsis cerisii* in the Greek Seas as, apart from a very old record from the south coast of Peloponnese (Guérin 1832), it has never been reported from the eastern Mediterranean. Finally, working on the molecular delimitation of stomatopods of the Greek Seas is necessary, in order to investigate the possible occurrence of cryptic species in future studies.

**Figure 1.**
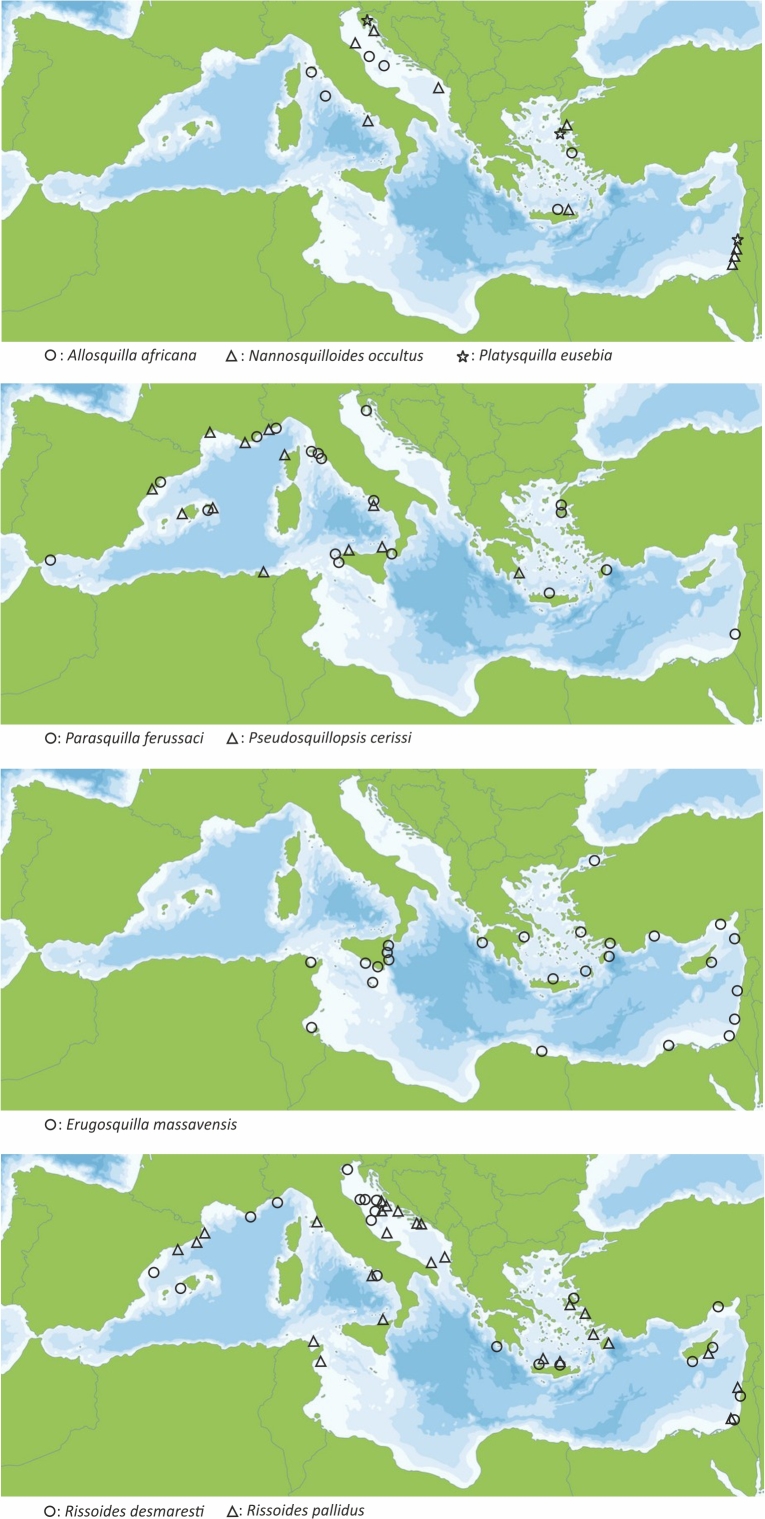
Distribution of eight out of the nine Stomatopoda species listed in this work in the Mediterranean Sea.

The distribution of the eight out of the nine Stomatopoda species of the Greek Seas (excluding *Squilla mantis* which is extremely common and widely distributed) across the Mediterranean basin is presented in Fig. 1. The only erythrean species *E. massavensis* present in the Greek waters (Saronikos Gulf, Crete, Rhodes, Karpathos and Zakynthos islands) until now has also been recorded along the coasts of Turkey, Syria, Cyprus, Lebanon, Israel, Egypt, Libya, Sicily, Malta, Tunisia and even from the Sea of Marmara (Shakman and Kinzelbach 2007, Özcan et al. 2008, Özcan et al. 2014, Ounifi-Ben Amor et al. 2015, Corsini-Foka et al. 2017, Zenetos et al. 2018, Gianguzza et al. 2019, Stern et al. 2019).

## Supplementary Material

XML Treatment for Stomatopoda

XML Treatment for Nannosquillidae

XML Treatment for Allosquilla africana

XML Treatment for Nannosquilloides occultus

XML Treatment for Platysquilla eusebia

XML Treatment for Parasquillidae

XML Treatment for Parasquilla ferussaci

XML Treatment for Pseudosquillopsis cerisii

XML Treatment for Squillidae

XML Treatment for Erugosquilla massavensis

XML Treatment for Rissoides desmaresti

XML Treatment for Rissoides pallidus

XML Treatment for Squilla mantis
